# Current Status of microRNA-Based Therapeutic Approaches in Neurodegenerative Disorders

**DOI:** 10.3390/cells9071698

**Published:** 2020-07-15

**Authors:** Sujay Paul, Luis Alberto Bravo Vázquez, Samantha Pérez Uribe, Paula Roxana Reyes-Pérez, Ashutosh Sharma

**Affiliations:** Tecnologico de Monterrey, School of Engineering and Sciences, Campus Queretaro, Av. Epigmenio Gonzalez, No. 500 Fracc. San Pablo, Querétaro CP 76130, Mexico; a01208914@itesm.mx (L.A.B.V.); a01702695@itesm.mx (S.P.U.); a00828207@itesm.mx (P.R.R.-P.)

**Keywords:** MicroRNA, neurodegenerative disorders, miRNA therapeutics, miRNA delivery

## Abstract

MicroRNAs (miRNAs) are a key gene regulator and play essential roles in several biological and pathological mechanisms in the human system. In recent years, plenty of miRNAs have been identified to be involved in the development of neurodegenerative disorders (NDDs), thus making them an attractive option for therapeutic approaches. Hence, in this review, we provide an overview of the current research of miRNA-based therapeutics for a selected set of NDDs, either for their high prevalence or lethality, such as Alzheimer’s, Parkinson’s, Huntington’s, Amyotrophic Lateral Sclerosis, Friedreich’s Ataxia, Spinal Muscular Atrophy, and Frontotemporal Dementia. We also discuss the relevant delivery techniques, pertinent outcomes, their limitations, and their potential to become a new generation of human therapeutic drugs in the near future.

## 1. Introduction

**Figure 1:**
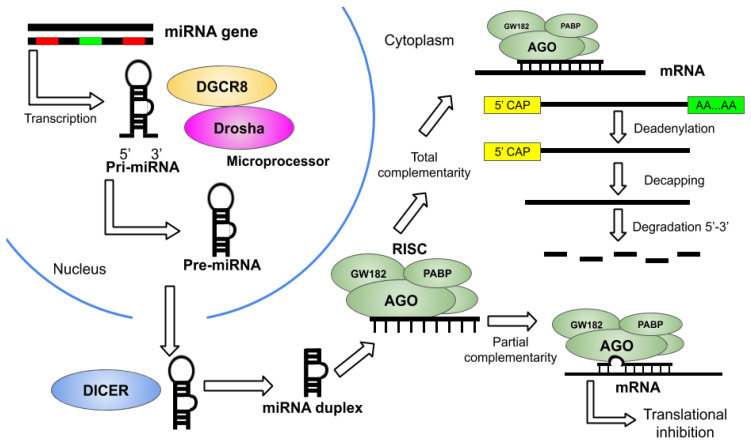
Scheme of miRNA biogenesis and the mode of action of RISC. The pri-miRNA is obtained in the nucleus by the translation of a specific gene and then transformed into pre-miRNA by the microprocessor complex conformed by DGCR8 and Drosha. Afterward, it is transported to the cytoplasm, where the DICER protein removes the loop of the molecule resulting in the miRNA duplex, thereafter, it joins the RISC by the AGO protein. When the RISC has total complementarity, the mRNA target is deadenylated, decapped, and completely degraded. Otherwise, partial complementarity can reduce or inhibit the translation and eventually cause mRNA degradation.

MicroRNAs (miRNAs) are small non-coding RNA molecules that regulate post-transcriptional gene expression by directing target mRNA cleavage or translational inhibition. The biogenesis of the miRNAs is conducted by the DROSHA and DICER RNAse III enzymes. At first, the process takes place in the nucleus, where the microprocessor complex formed by DROSHA, DGCR8 and associated proteins produce precursor miRNAs (pre-miRNAs) that are subsequently transported to the cytoplasm where the DICER enzyme processes them to form miRNA duplexes which are incorporated later to the RNA-induced silencing complex (RISC) [1,2,3] (Figure 1).

It has been predicted that more than one third of human genes are regulated by miRNAs; thus, they are involved in various cellular processes such as cell differentiation, cell proliferation, cell death and in the immunological responses associated with pathological and physiological problems [2]. For instance, miR-9, miR-134, miR-137, miR-124, miR-79, and miR-132, to name a few, are involved in neurodevelopment, neuroplasticity, synapse, and dendrite spine formation [4,5]. In addition to miRNAs, RISC contains multiple proteins such as Ago2, SND1, AEG-1, FMR1, VIG, and R2D2, which also regulate the proteins that are related to the control of pathophysiologies [6].

Neurodegenerative Diseases (NDDs) are chronic progressive disorders of the nervous system that generate negative impacts on neurological and behavioral function, implying biochemical changes that induce distinct clinical syndromes and histopathologies [7,8]. Gradual dysfunction of synapses, neurons, and glial cells, dementia, cognitive decline, brain function variations, and movement disorders are the early indicators of NDD [9]. The principal neurodegenerative diseases are Alzheimer’s disease (AD), Parkinson’s disease, amyotrophic lateral sclerosis (ALS), and Huntington’s disease [8]; however, Friedreich’s Ataxia (FRDA), Spinal Muscular Atrophy (SMA) and Frontotemporal Dementia (FTD) are also very important neurodegenerative diseases that are often ignored.

Dysregulation of miRNAs may promote neurological deterioration triggering the development of NDDs; thus, replacing or inhibiting downregulated or upregulated miRNAs in NDD subjects may be clinically beneficial [10]. With that rationale, miRNA-based therapeutic approaches have emerged as a promising alternative for treating NDDs; however, for this purpose, development and improvement of synthetic miRNAs along with specific and effective delivery systems are needed [11].

In the present review, we address a general inspection of some of the most studied miRNA dysregulations related to the onset and progress of NDDs, and the novel miRNA-based clinical approaches have been taken so far to combat those diseases. Nevertheless, the limitations and challenges of this technology are also discussed in this review.

## 2. Overview of miRNA Therapeutics

Since the discovery of miRNAs in *Caenorhabditis elegans* (1993), and their subsequent observation in human cells [12], the research interest in this field has grown rapidly with the aim of understanding their underlying implications in human metabolomics. Regarding neurodegenerative disorders, the miRNA dysregulation generated by alterations in the miRNA expression or biogenesis promotes neurodegeneration [13].

We can compile miRNA-based therapeutic approaches into two fundamental groups: miRNA inhibition to diminish the expression of disease-induced miRNAs and miRNA replacement to reestablish the expression of disease repressed miRNAs [14]. Into the bargain, miRNA-based therapeutics are being studied, since these RNA molecules provide different advantages. At first, one single miRNA has the ability to down-regulate several targets by joining the 3′ prime untranslated regions (3′UTR) of a gene. On the other hand, its size of ~22 nucleotides facilitates the design of miRNA drugs. Moreover, they can be delivered by multiple drug delivery systems that have already been authorized for human use [2]. Nowadays, the use of miRNAs for therapeutic purposes faces many challenges. Despite the fact that the use of special software has allowed the identification of human miRNA target genes (MTGs), the precision rate of this method is about 26% [15], because of that, it is of the utmost importance to check whether the predicted miRNAs really fulfill their function in vitro and in vivo; otherwise, they could be unsuitable for therapeutic purposes [16].

Moreover, determining which miRNAs could be potentially used to treat neurodegenerative diseases requires orderly investigation progress. According to Campbell and Booth [17], firstly, those miRNAs that show abnormal expression levels in a specific disease are associated with the regulation of that disorder, and later, those levels are provoked in a model of the disease to understand their role, but the unpredictable behavior of the miRNAs could show varied data for a single disorder, and the process of obtaining tissue samples presents difficulties. Owing to that, progress in the study of miRNAs in neurodegenerative diseases has been slow. Once the miRNA candidate is recognized, its target genes and biological functions must be studied both theoretically and experimentally. Finally, cell model systems and animal models induced with the corresponding disease should be treated with miRNA mimics or inhibitors to test the effects of the miRNA in the progression of the disorder.

**Figure 2:**
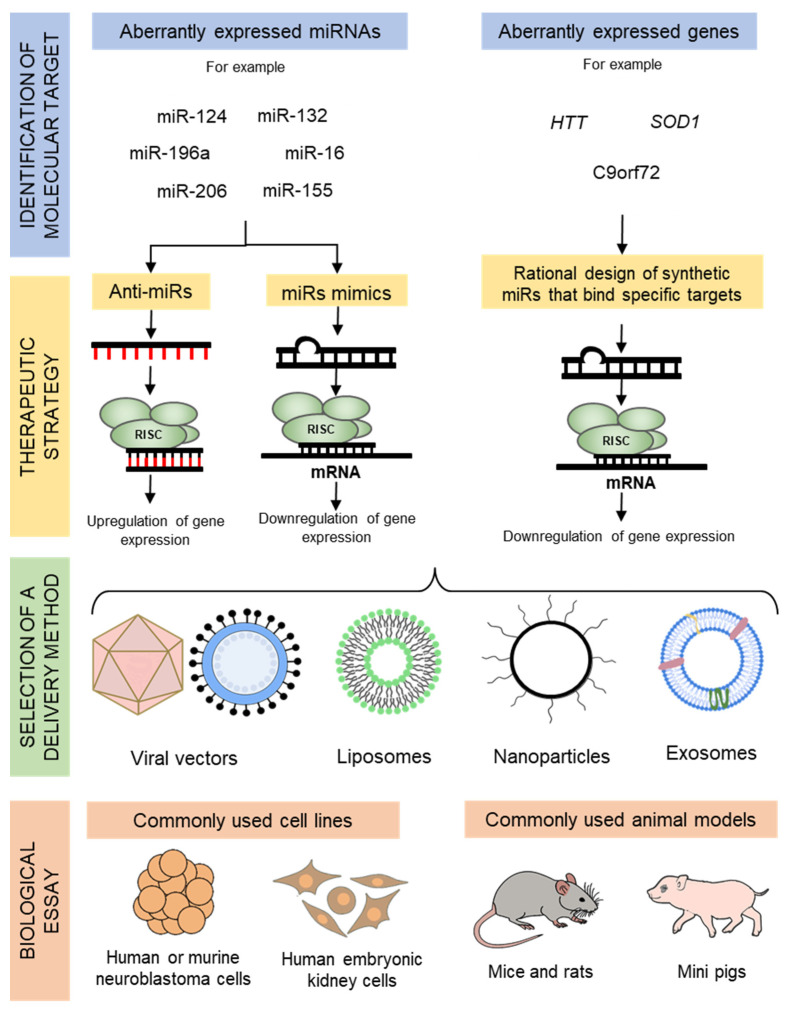
The general flow for designing and testing a miRNA-based therapeutic approach to treat neurodegenerative diseases. Firstly, abnormally expressed miRNAs are detected in patients’ tissues, and are then later regulated by inhibiting or enhancing their function. Otherwise, specific synthetic miRNAs can be designed to target genes of interest. Later, these miRs must enter cells by an appropriate delivery system. The effect of administered miRNAs is then assessed in biologic models.

MiRNA mimics are artificial miRNA duplexes that work as their natural mature counterparts; hence, they do not need to be processed and they directly join the RISC to interact with their respective mRNA targets and produce an effect on the phenotype of an organism [18,19]. On the other hand, artificial miRNA inhibitors are fully or partially complementary oligonucleotides that trap miRNAs and inhibit their functions, thus allowing the expression of various genes. They are synthesized either by genetic methods or by chemical processes [20] to test their effects in vitro or in vivo. The most common inhibitors are anti-miR oligonucleotides, which are commonly designed to block the activity of their target miRNA by making a perfect match with it [21] and the respective target transcript can be sequestered [22]. The general rationale for designing, developing, and testing miRNA-based therapeutics to treat NDDs is depicted in Figure 2.

## 3. Delivery Techniques for miRNA Therapeutics

One of the biggest concerns within gene-based therapy is the delivery of the therapeutic agent to the intended place, which is obligated to surpass the biological barriers without undergoing degradation in the bloodstream or renal excretion [23]. Thus, for the delivery of modified and unmodified miRNA molecules, specific carriers have been developed employing viral and non-viral systems, aiming to effective biocompatible and biodegradable devices [24].

Viral vectors are regularly used to deliver genetic material. Retrovirus, adeno-associated virus (AVV), and lentivirus are the most used. The main advantages of this system include prolongation of substitution or suppression of miRNA [25], repression of replication, and high efficiency in transfection processes [26]; still, the efficiency rate could be affected due to immune responses generated after virus delivery [27]. In retroviral vectors, the process for incorporating DNA into host chromosomes involves the reverse-transcription of viral RNA, which takes place at the cytoplasm; retroviruses can only be used in dividing cells. Lentiviruses derive from retroviruses and are widely used in the treatment of NDD, since they can infect both dividing and non-dividing cells [26]. AAVs can also infect these cells, but in order to replicate, the existence of a helper virus is essential [28]; AVV transfers smaller genetic sequences in comparison with lentiviruses (4.8 KB and 8 KB, respectively), and thus they are appropriate for miRNA transfection; nevertheless, AVV could trigger deterioration of tissues and inflammatory responses since they produce toxin [26].

Despite not having such high transfection efficiency as viral systems, nonviral systems allow less toxic transfection without limitations due to DNA size [26]. Many materials have been used to engineer non-viral delivery systems. Liposomes are widely used nonviral systems for transfection of miRNA mimics or anti-miRs in in vitro systems [29]; nonetheless, undesired immune responses and toxicity hinder safe delivery [30]. Its composition is similar to that of the cell membrane [31]; inside, nucleic acids are encapsulated. Depending on the charges, liposomes are subdivided into neutral, anionic, and cationic types. The cationic types of liposomes are made of cationic and Polyethyleneglycol (PEG) lipids, in addition to helper lipids, which increment the stability and minimize their toxicity [32]. Because of their characteristics, which include elevated compatibility with the cell membrane and facility of production, cationic liposomes are the most frequently used system; however, their short lifespan represents their main disadvantage [26]. Modifications with PEG could produce more stable complexes, in addition to several improvements [33]. Lipid nanoparticles (LNPs) have been used for in vivo delivery of oligonucleotides; this is feasible due to the electrostatic interactions that are formed between them [34]. LNPs are conformed by a PEG moiety and 1,2-dioleyl-3-dimethylammonium propane or DODAP, which is an ionizable cationic lipid [35]. The enhanced encapsulation efficiency and the higher stability of the vesicle make LNPs a more suitable system than liposomes [36]. On the other hand, to avoid chemical modifications, the encapsulation of both miR mimics and anti-miRs in nanoparticles (NPs) has already been accomplished. NPs have multiple advantages for in vivo delivery, including preservation payload of miRNA agents, improvement of target specificity and tissue distribution; however, encapsulation efficiency could be reduced since NP are highly hydrophilic [29]. Extracellular vesicles (EVs) refer to exosomes and microvesicles; it has been demonstrated that EVs can transport microRNAs and other components to the target cell [37] and they act as natural vehicles with minimal adverse effects. In contrast with liposomes, EVs could represent a better system, since it is initially necessary to synthetize structures of the cell membrane that EVs already possess [36].

## 4. Alzheimer’s Disease (AD)

Alzheimer’s Disease (AD) is a progressive, age-dependent neurodegenerative disease characterized by the accumulation of β-amyloid plaques and hyperphosphorylation of tau proteins, which induce the development of neurofibrillary tangles [38,39], resulting in a gradual decrease in cognition and short-term memory [40]. Patients show memory, judgment, orientation and language impairment, mood swings, and hallucination. Advanced age and family history are risk factors. Several genes have been linked with AD, including Amyloid-beta precursor protein (*APP*), Collagen, type XXV alpha 1 chain (*COL25A1*), Bromodomain PHD finger transcription factor (*BPTF*) and Caspase 2 (*CASP2*) [38]. Numerous studies have proposed that dysregulation of microRNAs impact on the pathophysiology of the disease; thus, they could represent a novel therapeutic approach to prevent or even stop AD progression [41].

In 2015, Parsi et al. [42] conducted an experiment in which overexpression of miRNA-16 was achieved in mice brains through the delivery of oligonucleotide using an osmotic pump, resulting in downregulation of *Gfap* an *Aif1*, providing neuron protection and oxidative stress prevention, respectively; while an in vitro experiment using cell transfection to up-regulate miR-16 ended up in a reduction of Tau phosphorylation, *BACE1* and *APP* genes. Likewise, in Neuro2a cells, reduction in tau protein expression was observed after being transfected with miR-132 mimics, which were later introduced in mice by osmotic pumps to enhance long-term memory [43]. Similarly, intracerebroventricular ICV injections containing oligonucleotides of miR-132 mimics were applied in mice; resulting in amplified levels of inositol 1,4,5-trisphosphate 3-kinase B (*ITPKB*), linked with tau phosphorylation and β-Amyloid Peptide (Aβ) deposition [44].

Analogously, it has been shown that overexpression of miR-124 via cell transfection in PC12 cells (a cell line derived from a pheochromocytoma of the rat adrenal medulla) and primary culture of hippocampal neurons, prevent cell damage and lower *BACE1* expression, which is involved in β-amyloid peptide production [45]. Additionally, PC12 cells along with SH-SY5Y cells were transfected with miR-193a-3p mimics, inhibitor, and negative controls; the overexpression of the miRNA, which targets *PTEN* gene, was able to reduce neurotoxicity produced by β-Amyloid [46]. Sun et al. [47] transfected PC12 cell with miR-107 while simultaneously subjecting them to 6-hydroxydopamine (6-OHDA), a neurotoxin known for inducing motor abnormality; results exhibited a decrease in Caspase-3 activity, a protein linked with apoptosis, along with repression in LDH release (that occurs after cell damage) and the elevation of SOD to reduce oxidative stress associated ROS activity. This study identified programmed cell death 10 (PDCD10) protein as a target of miR-107. Moreover, in a consequent in vivo assay, tail vein injection of a recombinant lentivirus expressing miR-107 was applied to mice treated with 6-OHDA, resulting in the up-regulation of miR-107 and consequently repressing 6-OHDA effects. The same delivery mechanism was used by other workers to introduce miR-326 lentiviral vectors into mice, and after analyzing the brain tissue, it was observed that overexpression of miR-326 was favorable, since it inhibited JNK signaling pathway, leading to an improvement of cognitive function; accumulation of Aβ was reduced and *VAV1* protein expression was restrained [48].

Apart from this, hippocampal injections of miR-34c inhibitors restored *SIRT1* levels, a protein that contributes to memory impairment when absent [49]; intranasal delivery of the aforementioned miRNA antagomir reversed the deterioration of dendritic spines caused by β-Amyloid (Aβ) protein [50]; this was equally achieved after miR-188-5p oligonucleotide transfection into primary hippocampal neurons from 5XFAD mice [51]. Recently, Zolochevska et al. [52] explored the impact of three different miRNAs (miR-149, miR-485 and miR-4723) in resilience against Aβ oligomers, which may produce synaptic dysfunction after interacting with the synapses; in vivo experiments where miRNAs were introduced via ICV injection in both male and female mice suggested that all the selected microRNAs could prevent Aβ oligomer binding. Interestingly, it was detected that target genes and RNA regulation are sex-specific. Likewise, ICV injections were used to transfect miR-200b and miR-200c mimics into mice; primary murine neuronal cells and SH-SY5Y cells were equally transfected; results suggest that both miRNAs could prevent toxicity derived from Aβ; overexpression of these miRNAs in mice resulted in models achieving remembering of a particular location [53]. Overexpression of miR-31 in the hippocampus of AD triple-transgenic mice after stereotaxic injection of lentiviral particles encoding the primary miRNA, reduced *APP*, and *BACE1* protein expression, where the latter promotes sequential processing of the former, resulting in accumulation of Aβ, recovery of cognitive functions, improved memory, decreased anxiety levels and reduced intraneuronal Aβ [54]. In the same way, overexpression via cell transfection of miR-16 in Neuroblastoma2a and NIH3T3 murine cells and infusion into a lateral ventricle in mice led to a reduction of *APP* levels in the hippocampus [55]. Cell transfection of miR-206 antagomir in primary hippocampal neurons, Neuro-2a, and SH-SY5Y cells provoked a rise in Brain-Derived Neurotrophic Factor (*BDNF*) protein levels; these results were replicated in mice after intraventricular injection and intranasal delivery of the same antagomir [56]. Inhibition of the miRNA miR-592 in the brain tissue of male rats up-regulates the level of the *KIAA0319* gene, which triggers the Keap1/Nrf2/ARE signaling pathway and stimulates viability of astrocytes and lowers oxidative stress injury [57]. An additional experiments, overexpressing miR-101 via cell transfection in HeLa U373 and PC12 cells found that *APP* levels were reduced too [58].

## 5. Parkinson’s Disease (PD)

Parkinson’s Disease (PD) is a progressive neurological disorder in which patients display symptoms of rigidity, tremor, postural instability, and bradykinesia. Moreover, it is pathologically distinguished by the declination of the dopaminergic neurons located in the nigrostriatal and by the manifestation of the misfolded α-synuclein in the surviving neurons [59]. However, different studies indicate that multiple genes influence the risk of developing PD, especially *SNCA*, *LRRK2*, and *GBA*. *GBA*, the most studied among them, encodes glucocerebrosidase enzyme, whose loss of function is related to the diminution in the degradation of the α-synuclein produced by *SNCA*, resulting in the formation of Lewy bodies [60]; on the other hand, *LRRK2* is correlated with microtubule-binding, vesicular transport, and autophagy, and its pathogenic variants produce an augment in both phosphorylation and cell death; thus kinase inhibitors have a potential role in the treatment of PD [61]. Therefore, diverse investigations have examined the potential role of the miRNAs silencing the genes of interest. Case in point, a study in which the effects of miR-205 were analyzed in brains of sporadic PD patients, cell lines, primary neuron cultures, and mouse midbrain dopaminergic neurons proved that it can drop the expression of the LRRK2 protein owing to a conserved binding site located in the untranslated region of the 3′ extreme of the *LRRK2* gene and prevent the defects of the neurite outgrowth [62].

Khodr et al. [63] tested the effect of mir30-hSNCA delivered by AAV2/8 in a rat model and noticed that the silencing of the *SNCA* gene caused by this miRNA protected against the deficit in the forelimb and decreased the loss of tyrosine hydroxylase-immunoreactive (TH-IR) neurons in the substantia nigra (SN), but unfortunately negative outcomes like toxic effects in the striata, absence of total protection of TH-IR and swelling in the SN were noticed, and hence the idea of using the dose applied in this experiment for clinical PD therapeutics was dropped. The role of miR-30e in a 1-methyl-4-phenyl-1,2,3,6-tetrahydropyridine (MPTP) induced PD mouse model was examined, concluding that it downscales the neuroinflammation by lowering the activity of the NLRP3 protein, besides, an attenuation in the loss of dopaminergic neurons was described in the pars compacta of the substantia nigra (SNpc), accompanied by a reduction in the expression of the α-synuclein [64]. In addition, the impact of miR-124 was studied in 1-methyl-4-phenylpyridinium ion (MPP^+^) intoxicated SH-SY5Y cells and in a MPTP intoxicated mouse model of PD, resulting in the suppression of the BIM protein thus mitigating the autophagy, apoptosis and lysosomal exhaustion. The neuroprotective mechanism of miR-124 could be based on the suppression of the Bax translocation to mitochondria associated with the silencing of *BIM* [65]. Similarly, other studies revealed that miR-124 loaded in biocompatible nanoparticles (NPs) boosted the neuronal maturation and proliferation of neural stem cells and neuroblasts in vitro, and augmented the number of neuroblasts that can migrate to the cell layer belonging to the olfactory bulb and the damaged striatum in 6-hydroxydopamine (6-OHDA) mice models of PD, improving the symptoms of those mice [66,67].

A study in human MPP^+^-induced SK-N-SH neuroblastoma cells indicated that the transfected miR-181a is potentially associated with the regulation of autophagy and apoptosis in PD due to the inhibition of the p38 MAPK and JNK signaling pathways [68]. Another study on MPTP-treated SH-SY5Y cells demonstrated that the overexpression of transfected miR-185 also clogs both apoptosis and autophagy in dopaminergic neurons by the suppression of the AMPK/mTOR intracellular signaling pathway [69]. In contrast, Gao et al. [70] examined the effect of miRNA-183 delivered by cell transfection in a culture of SN neurons from MPTP-treated PD mice, concluding that its overexpression causes the apoptosis of those neurons because of the inhibition of *OSMR* expression and they suggested that PI3K-Akt signaling pathway could be the mechanism related to this process.

To further investigate the impact of miR-155, a mouse model with an entire genetic deletion of this miRNA was analyzed, and microglial cells were treated with an oligonucleotide mimic of miR-155. The results indicated that this miRNA is involved in the inflammation promoted by the α-synuclein, besides, from the neurodegeneration caused by this protein. These outcomes were related to the induction of the expression of the major histocompatibility complex class II (MHCII), which was blocked by the suppression of miR-155 [71]. Similarly, it was ascertained that the miR-7 controls the inflammation produced by the nod-like receptor protein 3 (NLRP3), additionally, it decreased the degeneration of dopaminergic neurons and the microglial activation in a PD model of MPTP-induced mice [72]. Another investigation on the impact of miR-494-3p in MPP^+^-induced SH-SY5Y cells implied that the inhibition of miR-494-3p downscales the neurotoxicity caused by MPP^+^ and allows the manifestation of SIRT3, a protein that takes part in metabolic control, aging and deacetylation of mitochondrial enzymes. It was also observed that the MPTP-induced PD mice presented motor deterioration after a treatment with miR-494-3p, which prevented the expression of SIRT3 [73,74]. Wang et al. [75] explored the effect of miR-9-5p transfected into MPP^+^-intoxicated SH-SY5Y cells, observing that its knockdown mitigated the neurotoxicity caused by MPP^+^ and that the overexpression of miR-9-5p decreases the expression of SIRT1 protein, whose main role is the regulation of the deacetylation of several transcription factors and could be involved in the relief of neurotoxicity. Moreover, there was a decline in oxidative stress, apoptosis, and inflammation.

A recent research demonstrated that the suppression of the expression of miR-96 caused by an inhibitor in a PD mouse model induced via MPTP stops the manifestation of Inducible nitric oxide synthase (iNOS) and impedes the apoptosis of the dopaminergic neurons by fostering the activity of *CACNG5*, resulting in blocking the MAPK signaling pathway. These results were later confirmed in SH-SY5Y cells; furtermore, the cell culture presented an increase in nigral cells [76]. Into the bargain, Yang et al. [77] examined the impact of miR-216a transfected into MPP^+^-induced SH-SY5Y neuroblastoma cells, observing that it decreases the neurotoxicity provoked by MPP^+^ and lowers neuronal apoptosis owing to the fact that it targets Bcl-2-associated X protein (Bax), whose main role is the regulation of apoptotic and anti-apoptotic processes. In recent years a great deal of research has been done related to miRNA-based therapies for PD and their positive results imply that clinical trials may begin soon.

## 6. Huntington’s Disease (HD)

Huntington’s Disease (HD) is a neurodegenerative disorder of the central nervous system that is distinguished by psychiatric and behavioral alterations, undesired motions, and dementia [78], and caused by abnormal CAG repeat expansion in exon 1 of the Huntingtin gene (*HTT*) [79]. The aforementioned mutation results in mutant Huntingtin (mHTT) protein, whose accumulation causes neuronal dysfunction and death [80]. Since this mutation occurs in a specific locus, it can be treated with gene therapy by using non-coding small RNA to decrease or avoid the translation of the mRNAs targets into their correspondent protein [81]. In various early investigations, the silencing of the mutant *HTT* with miRNA has been analyzed, but the procedures also attenuated the expression of the normal gene, and although studies in macaques and mice have proved that the nonallele-specific mutant *HTT* silencing could work safely and efficiently [82,83], there is not enough information as to whether the neural cells of the brain will resist the silencing of both alleles in a long-range way [84]. Nevertheless, new methods are being developed to target specifically the mutant allele with miRNA.

Monteys et al. [84] designed artificial miRNA molecules to target mutant *HTT* and after the transfection of the artificial miRNAs into HEK293 cells, preferential silencing of the mutant allele over the wild type (WT) was observed; however, the normal one was still affected. It was also observed that when the amount of miRNA supplied increases, their allele preference gets reduced. In addition, the authors used the artificial miRNAs: mi306.12v16G and mi268.5 in transgenic mice that had the alleles of interest, but the expression of both was similar, showing that, in the case of the mi268.5, there was no specific silencing and that mi306.12v16G targeted more at the mutant HTT allele, with this outcome validating its selectivity. Similarly, the effect of miR-27a in the reduction of the aggregation of mHTT in derived neuronal stem cells was examined, finding a rise in the performance of the MDR-1 protein, which helped to decrease the conglomeration of mHTT. These results buttress that the miR-27a downscales the mHTT accumulation by stimulating the function of the MDR-1 [85].

Moreover, it was noticed that an increased expression of miR-196a produced by transfection diminished the manifestation of the mHTT in human embryonic renal cells and neuroblastoma cells from mice [86]. Additionally, this miRNA avoided the expression of mHTT by suppressing its synthesis, specifically using a protein degradation process [86]. Furthermore, the influence of miR-196a was tried out in transgenic mice models and HD individual-derived iPSC (HD-iPSCs), resulting in staving off the manifestation on mHTT in vivo and in vitro, thus supporting that it can help in developing therapies for humans [86]. On the other hand, the role of miR-196a was examined in cultured primary cortical neurons and miR-196a overexpressing transgenic mice, in both models the neurons presented a larger neurite length and more limbs, these outcomes demonstrated the effect of miR-196a in the differentiation and morphology of neuronal cells both in vitro and in vivo by the suppression of the expression of RANBP10, a protein that affects neuronal functions and morphology. The outcomes were also related to the improvement of the β-tubulin polymerization [87].

Fukuoka et al. [88] studied the effect of the miR-132 in the brain of HD mice, where the miRNA was delivered using an adeno-associated virus (AAV9-miR-132). Their results documented three important advances: firstly, the target of the miR-132 could be *MECP2*; secondly, the symptoms of the HD mice were ameliorated; and finally, the treated mice had a more extended life when comparing them with the controls. In this investigation, there was no considerable change in the expression of mHTT, so the miR-132 could not work as a silencer of the gene at issue. On the other hand, an examination of the effect of exosomal delivery of the miR-124 performed in the striatum of HD transgenic mice revealed the lower manifestation of the REST protein in the treated mice. Given that miR-124-REST is involved in neurogenesis, the level of Doublecortin (DCX) protein was assessed, but no significant difference was found. However, the treatment did not produce any improvement in the behavior of the treated mice [89]. Another investigation found that miR-124 increases the neurogenesis in the cortex and striatum of transgenic HD mice, establishing the possible attenuation of the development of HD. Augmented neuronal survival and the enhancement of the mice behavior were also observed [90].

The impact of miR-22 was observed in primary cultures of cortical and striatal neurons infected by lentiviruses that induced the manifestation of WT human HTT, human mHTT, and miR-22, showing that this miRNA is associated with different anti-neurodegenerative processes including apoptosis inhibition, regulation of the expression of genes related to apoptosis (*MAPK14*, *Trp53inp1*), and HD (*Rcor1*, *HDAC4*, and *Rgs2*); additionally, it decreased the conglomeration of mHTT, but the process through which that diminution occurred remains unclear. Altogether, these results point out miR-22 as a potential therapeutic element for HD treatment [91].

Recently, in a study, an artificial miRNA was created using miR-155 as a template and was delivered by an AAV injection (AAV9-U6-miR^HTT^) into the striatum of transgenic HD sheep that expressed the human HTT completely with the CAG repeated 73 times. The amount of human mHTT was reduced in the neostriatum, putamen, and caudate without affecting the sheep’s own Huntingtin. These authors proposed to develop a method for selectively inhibiting the expression of the allele of importance by using various AVV-miRs designed to target a specific nucleotide [92]. Additionally, different analyses have been carried out to test the effect of constructs of miRNAs designed to target specific genes and delivered by the AAV5 vector in HD models. Miniarikova et al. [93] noticed that after injecting various of those constructs into the brain of HD rats, the accumulation of mHTT was reduced and the neuronal dysfunction related to DARPP-32 was lowered in the striatum, the AAV5-miHTT-451 construct was the one that showed the greatest impact. During this procedure, there were no signs of astrocytes or microglia, implying that there was no activation of an immune response against the AAV5-miHTT gene therapy, demonstrating that it could be a safe therapeutic method for treating HD. The efficacy of AAV5-miHTT was proved in a transgenic HD minipig model by an intracranial administration and the results indicated a high reduction of the human mHTT mRNA in the caudate nucleus, cortex, thalamus and putamen, regions of the brain that are linked with the HD. Owing to the fact that the endogenous porcine HTT levels were not affected during the treatment, these data demonstrate the specificity of AAV5-miHTT targeting human HTT [94]. Moreover, the effect of AAV5-miHTT was examined in HD mouse models, in this instance, the reduction of the conglomeration of HTT was lower than it was expected, but the mice manifested ameliorate in their functions, a rise in their survival median and bigger weights without the appearance of problems related to the tolerability of the treatment [95]. In the same way, AAV5-miHTT was tested recently in a humanized HD mouse model through intrastriatal administration, showing that it reduces the presence of HTT in the cortex and striatum; the treatment did not show any problems related to its tolerance in low doses [96]. These outcomes suggest that clinical trials of this miRNA-mediated gene therapy may begin to be applied soon.

## 7. Amyotrophic Lateral Sclerosis (ALS)

Amyotrophic lateral sclerosis (ASL) is a fatal, progressive, and incurable neurodegenerative disease characterized by selective degeneration of motor neurons in the brain and spinal cord that can draw death by respiratory failure in just 3 to 5 years. This disease commences with focal weakness, which later spreads to most muscles; once corticospinal motor neurons fail, muscle stiffness and spasticity occur; if motor neurons are affected, spontaneous muscle twitching and ultimately muscular atrophy might emerge [97]. Cell accumulation of misfolded or abnormal proteins is a hallmark in ASL but it is important to highlight those diverse genes and pathophysiological processes account to the disease, that can either occur in sporadic forms or have a positive familial history. Clinical manifestations of ASL often also include cognitive and behavioral impairment, and even collateral behavioral-variant frontotemporal dementia [98,99].

An early study performed in 2012 on leukocytes isolated from ASL patients identified eight dysregulated miRNAs (hsa-miR-338-3p, hsa-miR-45, hsa-miR-1275, hsa-miR-328, hsa-miR-638, hsa-miR-149, hsa-miR-665, and hsa-miR-583) [100], and from there on, copious investigations have explored cerebrospinal fluid (CSF), blood and muscle biopsies from ALS patients looking for miRNAs that could serve as biomarkers for ASL diagnosis [101]. For instance, a recent comprehensive analysis revealed at least 40 miRNAs differentially expressed in the muscle tissue of ALS subjects [102]. However, research on miRNAs in ASL has made a very expected jump into hunting miRNAs-based techniques that can provide a therapeutic alternative for this lethal disease. This often involves the use of specifically developed animal models, such as transgenic rodents expressing the most common genetic mutations in ASL: Superoxide dismutase 1 gene (*SOD1*) and repeat nucleotide expansions in the gene encoding C9ORF72 [103]. For instance, considering the significant mutation in ASL of the intronic G4C2 repeat in chromosome 9 open reading frame 72 (*C9orf72*) gene, believed to result in toxicity by an accumulation of RNA foci in the nucleus and deposition of dipeptide repeat (DPR) proteins in the cytoplasm. An experiment was designed to silence *C9orf72* employing miR-101 and miR-451 scaffolds (miC); consequently, two miC candidates were incorporated in a AAV5 vector in HEK293T cells and induced pluripotent stem cell (iPSC)-derived neurons, resulting in the silencing of C9orf72 which could help to alleviate the gain of toxicity described in ALS [104].

One case study initially detected an increased expression of miR-155 in ASL mice and human spinal cords, and so congruently later administered a miR-155 antagomir by ICV-directed osmotic pump and intraperitoneal injections to SOD1^G93A^ mice to assess its effects. The procedure slowed down ASL progression and resulted in prolonged survival of treated mice [105]. Likewise, it was identified that neurons under endoplasmic reticulum stress related to ASL showed increased miR-29a expression [106], which is known for targeting various genes including neuron navigator 3 (*NAV3*), b-site amyloid precursor protein cleaving enzyme 1 (*BACE1*) and genes encoding extracellular matrix components [107]. Following this discovery, the authors injected SOD1^G93A^ mice with miR-29a-specific antagomir via ICV, and despite progression and motor function not being significantly transformed, increased lifespan was detected in male mice [106].

An exciting prospect in miRNA-based therapy includes the rational design of synthetic RNA molecules to target specific genes and that can be illustrated with the consecutive experiments performed by Mueller and his team. In a first approach, a single-stranded AAV9 vector encoding an artificial microRNA against human *SOD1* (amiR*^SOD1^*) was engineered, employing endogenous murine miR-155 flanking sequences. The AAV9- amiR*^SOD1^* was then injected in neonatal *SOD1*^G93A^ mice cerebral lateral ventricles, resulting in an extended average survival and delayed hindlimb paralysis; furthermore, treated mice showed a reduction of mutant human *SOD1* mRNA levels and improvements in numbers of spinal motor neurons, the diameter of ventral root axons, and extent of neuroinflammation in the spinal cord [108]. Then, the research group reported the silencing of *SOD1* in adult *SOD1*^G93A^ mice with a method of delivery of artificial miRNA miR-*SOD1* by rAAVrh10 vector, resulting in delayed disease onset and death in the animal model, while conserving muscle strength and motor and respiratory functions. Furthermore, they proved their rAAVrh10-miR-*SOD1* system in marmoset, a non-human primate model, and assessed an effective and safe silencing of *SOD1* in lower motor neurons, this being one of the pristine studies of miRNA-based therapeutics for ASL in primates [109]. Finally, more recently, they presented a work where they injected *SOD1*^G93A^ mice in the tongue and intrapleural space with AAVrh10-miR*SOD1* system, showing diaphragm, tongue and systemic silencing of *SOD1*, which delayed disease onset and prolonged survival by approximately 50 days, which is the highest time achieved by the team to date. Furthermore, treatment delayed weight loss and limb weakness. Despite the therapy succeeding in improving breathing and survival, it failed to amend the restrictive lung phenotype [110]. Although we consider the AAV with artificial miRNAs targeting *SOD1* systems to show promising outcomes, we need to keep track of it for the next few years.

An innovative experiment was performed by Li. et al. that employed the well-established rAAVrh10 amiR-SOD1 system plus *GFP* as a reporter gene. In this experiment, they tested the effect of injection speed on transduction effectiveness in the central nervous system (CNS). *SOD1*^G93A^ mice were subjected to either slow or fast intrathecal (IT) injections; further analysis showed that both slow and fast IT injections were therapeutically effective, but slow injection presented improved outcomes, such as delayed onset and extended survival in treated mice. This suggests that when designing a miRNA-based therapy, the anatomical and physiological characteristics of CNS sites must be taken into consideration [111].

Altogether, these investigations show that in recent times, miRNA-based systems targeting ASL have successfully established their efficacy, and now are moving towards optimization in delivery.

## 8. Friedreich’s Ataxia (FRDA)

Friedreich’s ataxia (FRDA) is an autosomal recessive disease and a neurodegenerative disorder provoked by a trinucleotide repeat expansion in the first intron of the *FXN* gene [112] that reduces levels of frataxin protein [113], promoting oxidative stress. FRDA is the most recurrent form of hereditary ataxia [114]. The average age of onset in patients is 10.52 years old, and it equally affects male and female individuals. Early signs include scoliosis, foot deformity, and cardiomyopathy [115], while the most recurrent neurological signs are: ataxia of gait, clumsiness, dysarthria, finger–nose ataxia, dysdiadochokinesis, pyramidal weakness and sensory loss in the lower limbs [116]. Unfortunately, no cure or commercial therapy is available for this disease [112].

Limited miRNAs information linked with FRDA is available so far. Dantham et al. [113] identified six dysregulated microRNAs associated with FRDA, where has-mir-15a-5p and hsa-mir-26a-5p target the *BDNF* gene; hsa-mir-29a-3p, hsa-mir-23-3p and hsa-mir-223-3p target the Glucose-6-phosphate Translocase (*G6PT*) gene; hsa-mir-24-3p could regulate the Dihydrofolate Reductase (*DHFR*) gene and hsa-mir-21-5p could feasibly negatively regulate a fundamental regulator—peroxisome proliferator-activated receptor α.

Mahishi et al. [117], in 2012, evaluated the level of hsa-miR-886-3p and its implication on frataxin mRNA levels in the blood, where cell transfection using anti-miR-886-3p resulted in enhanced levels of frataxin mRNA. Nevertheless, the study was performed using unaffected cells, and later, it was pointed out that the small RNA miR-886-3p was no longer considered to be a miRNA; however, other authors continue to acknowledge it in the miRNA category [118]. The most recent work on miRNA therapeutics for FRDA has done by Bandiera et al. [4] by transfecting HEK-293 cells with hsa-miR-124 mimics, where the overexpression of the later was correlated with a down-regulated FRDA-3-UTR haplotype, which expands the FRDA risk haplotype to the 3′-UTR.

Undoubtedly, much more work is required to understand the biological mechanisms underlying miRNA regulation in FRDA, as well as novel in vitro and in vivo approaches that address whether miRNAs could be a suitable therapy for treating Friedreich ataxia.

## 9. Spinal Muscular Atrophy (SMA)

Spinal muscular atrophy (SMA) is an autosomal recessive, progressive disease that constitutes the most common genetic cause of death in children. It is distinguished mainly by the degeneration of α-motor neurons in the spinal cord. The identified cause of SMA is either a mutation or deletion in the survival motor neuron 1 (*SMN1*) gene, leading to skeletal muscle progressive atrophy, and later to symmetric limb paralysis, respiratory distress and, lastly, death [119,120]. Almost a decade ago, it was well-established that SMA presents a pathogenic mechanism caused by malfunctioning RNA metabolism and the biogenesis of microRNAs. In a SMA mice model, a correlation was found between motor neuron degeneration and down-regulation of miR-9, which targets heavy neurofilament subunit [121]. Since then, plenty of miRNAs have been acknowledged to have a role in SMA development, including miR-132, miR -206, miR -183, miR -335-5p, miR -431, miR -375, miR -2, mirR-218, miR-335-5p and miR-100-5p [122,123,124]. At least miR-9, miR-206, miR-183, and miR-375 have been pointed out to hold the potential to be explored as therapeutic targets for SMA [122]. However, only a few assays for testing the capacity of miRNAs over SMA features have been attempted. For instance, after finding that miR-146a was upregulated in SMA-induced pluripotent stem cell (iPSC)-derived astrocytes, the addition of the corresponding miR inhibitor resulted in protection from SMA astrocyte conditioned medium-induced toxicity in SMA iPSC-derived motor neurons [125].

Later, an experiment revealed that miR-431 regulates the neurite length of motor neurons by targeting genes related to motor neuron axon outgrowth, especially chondrolectin (Chodl) and transfection of motor neurons with anti-miR-431 increased Chodl expression by fully restoring both the total as well as the longest neurite length [126]. A very interesting approach was taken by Valsecchi et al. when they first established a direct linkage between the miR-206-HDAC4 (histone deacetylase 4) system and Fibroblast Growth Factor Binding Protein 1 (FGFBP1), involved in the regeneration of neuromuscular synapses after nerve injury. Cell transfection of miR-206 mimics lowered HDAC4 protein levels and resulted in FGFBP1 transcriptional up-regulation; as a result, the research team speculated that this miRNA had a neuroprotective mechanism [127]. Recently, they employed animal models to test this hypothesis by administrating miR-206 mimics via ICV to pups and mice and observed a reduction in the severity of SMA pathology, slower disease progression, increased lifespan, and improved behavioral performance. A deeper examination shows that miRNA-206 upregulation decreases sodium–calcium exchanger isoform 2 (NCX2), which regulates intracellular [Ca2+] and [Na+], conclusively suggesting that miR-206 might own part of its neuroprotective effect to modulation of NCX2 expression in SMA [128].

Despite the number of investigations for SMA, miRNA therapy is very limited; available studies show very exciting results that encourage the view of this genetic therapy as a promising alternative for the treatment of SMA in the future.

## 10. Frontotemporal Dementia

Frontotemporal Dementia (FTD) is a heterogeneous group of neurodegenerative disorders [129] characterized by disturbances in executive functioning, language, and behavior [130] and represents about 10% of existing dementias [131]. FTD is caused by the progressive neurodegeneration of both the anterior and frontal-temporal lobes [132]. It is estimated that more than the 50% of the occurrences of this disorder are produced by the accumulation of the 43 kDa TAR DNA-binding protein (TDP-43; FTLD-TDP); meanwhile, the rest of the cases are generated by the conglomeration of either the microtubule-binding protein tau (FTLD-tau) or the sarcoma protein (FTLD-FUS) [133]. It has been reported that mutations in *C9orf72*, *GRN* and *MAPT* genes are associated with FTD [129]; however, some mutations rarely occur in other genes, such as *TARDP*, *TBK1*, *VCP* and *CHMP2B* that are also related to FTD [134].

Piscopo et al. [135] identified several miRNAs that are altered in Frontotemporal Lobar Degeneration (FTLD), a term used to describe the aggregation of proteins in the temporal and frontal lobes, inducing their degeneration [133,136], and possesses pathways related to FTD. Those miRNAs are miR-132 and miR-212, whose target gene is *TMEM106B*; miR-107, miR-659, and miR-29b whose target gene is *GNR*; miR-9 targeting diverse genes implicated in neuronal specification and miR-124, the target the gene of which is *AMPAR* [135]. In addition, dysregulation of miR-922, miR-516a-3p, miR-571, miR-548b-5p, and miR-548 was found in samples from FTLD-TDP patients with mutations in the *PGRN* gene [137].

Nevertheless, studies about the therapeutic applications of miRNAs in the treatment of FTD are limited. Jiao et al. [138] analyzed the influence of miR-29b by transfecting NIH3T3 cells that expressed human progranulin (hPGRN, a protein whose mutation causes haploinsufficiency and is linked with FTD) as well as HEK293 cells, and their results showed that the expression of this miRNA lowers the manifestation of hPGRN in NIH3T3 cells and regulates the expression of endogenous hPGRN in HEK293 cells by targeting *GRN*. Furthermore, an investigation of the effects of miRNA-124 delivered by the injection of AAV-GFP-miR-124 in a mouse model of FTD with a mutation in the *CHMP2B^Intron5^* (*tTA: CHMP2B^Intron5^* mice) demonstrated that this miRNA downscaled the expression of *Gria2* and *Gria4* (subunits of AMPA receptors) in the medial prefrontal cortex of the *tTA: CHMP2B^Intron5^* mice and improved their social shortfalls; these results suggest that AMPA receptors are associated with the regulation of social behavior [139].

Additionally, Chen et al. [140] delivered miRNA-132 and miR-212 mimics through liposome transfection into HEK293 cells and both miRNAs reduced *TMEM106B* mRNA levels. Additionally, transfection of miR-107 into H4 and HeLa cells reduced the expression of *GRN* [141]; this effect was previously observed in M17 cells after transfection with miR-659 [142].

An overview of the current development status of miRNA-based therapeutics in NDDs is summarized in Table 1.

## 11. Conclusions

In recent years, extensive research has been performed to understand the molecular mechanisms behind the psychiatric afflictions such as AD, PD, HD, ALS, FRDA, SMA and FTD due to their complex etiology and the lack of effective treatments; and reports indicated that several inflammatory microRNAs are highly involved in the pathogenesis and pathophysiology of these NDDs.

Nonetheless, there are significant challenges that miRNA-based therapeutics are facing nowadays. Besides the aforementioned biological factors that could compromise the successful delivery of miRNAs, it is well established that not every relationship between miRNAs and their targets is one-to-one; rather, a single microRNA can have multiple targets, and this can generate undesirable side effects hindering therapy objectives [143,144]. Additionally, natural variability in the miRNAs expression patterns cannot be ignored. Recent studies have reported that the expression of miRNAs can be sex-biased, and thus sex-specific therapeutic strategies can be implemented in disease treatment [145]. Similarly, miRNAs expression is often influenced by age, and hence, longitudinal monitoring of patients over time must be carried out after the application of a miRNA therapy [146].

Although miRNA-based strategies hold the noteworthy potential extensive research and knowledge regarding the miRNA-target interaction during the development of the NDDs is highly required for the development of miRNA-based therapies in the near future.

## Figures and Tables

**Table 1:** Functional implications and therapeutic potential of different miRNAs associated with important neurodegenerative disorders.

Disorder	Targeted MiRNA	Antagonist or Mimics	Target Gene	Delivery Method	Biological Model or Tissue	Functional Implication	Reference
Alzheimer’s Disease (AD)	miR-16	Mimics	APP, BACE1	Liposomes	HEK293 cells	Reduced expression of BACE1 and APP	[42]
	miR-16,15,195				HEK293 cells overexpressing APP Swedish mutation	Suppression of Aβ production	
	miR-16				Neuro2a cells	Reduction of BACE1 and APP protein expression, decrease in Tau phosphorylation	
					HT22 cells	Reduction of BACE1 and APP protein expression	
			APP, BACE1, Tau	Oligonucleotide	Mice	APP downregulated in cortex, brainstem, and striatum. BACE1 reduced in the hippocampus, brainstem, and striatum. Tau downregulation in the hippocampus, brainstem, and striatum. ERK1 downregulated in the hippocampus and cortex. Nicastrin, Gfap, and Aif1 downregulation	
	miR-132	Mimics	Tau	Liposomes	Neuro2a cells cotransfected with a reporter construct containing mouse 3’UTR	Downregulation of tau expression	[43]
					Neuro2a cells		
				Cell transfection	3xTg-AD mice	Improve long term memory and reduction of phosphorylated tau	
	miR-132	Mimics	ITPKB	Oligonucleotide	Mice	Amelioration of amyloidosis and Tau hyperphosphorylation	[44]
	miR-124	Mimics	BACE1	Oligonucleotide	Hippocampal neurons, PC12 cells	Reduction of BACE1 expression and Aβ production	[45]
	miR-193a-3p	Mimics	PTEN	Liposomes	PC12 cells, SH-SY5Y cells	Attenuation of β-amyloid induced neurotoxicity	[46]
	miR-107	Mimics	PDCD10	Liposomes	PC12 cells, SH-SY5Y cells	Increase of cell viability, Reduction of Caspase-3 activity, LDH release, and ROS levels, increase SOD levels. Reduction of induced cytotoxicity	[47]
				Lentivirus	6-OHDA induced mouse model	Suppression of 6-OHDA induced motor disorder	
	miR-326	Mimics	VAV1	Lentivirus	Mice	Improvement in cognitive function, inhibition of JNK signaling pathway, decrease in Aβ deposition, inhibit protein expression of VAV1	[48]
	miR-34c	Inhibitor	SIRT1	Hippocampal injection	C57Bl/6J mice	Enhance memory, reinstate learning behavior and SIRT1 levels	[49]
	miR-34c	Inhibitor	SYT1	Intranasal administration	SAMP8 mice	Increase levels of SYT1, amelioration of cognitive function, enhance memory function, recovery of dendritic spine density	[50]
	miR-188-5p	Mimics	NRP2	Oligonucleotide	Primary hippocampal neurons from 5XFAD mice	Reverse synaptic damage induced by Aβ _1-42_ and reduction in dendritic spine density, enhance cognitive function impairments	[51]
	miR-149	Mimics	APP, BACE1, Tau	Cell transfection	SH-SY5Y cells	Resilience to Aβ oligomer	[52]
	mir-485		BACE1, Tau				
	miR-485		APP, Syn1, Ppp3ca, Mapt, Snap25, Snca, DNM1	ICV	C57B6 mice	Upregulation of APP, Syn1, Ppp3ca, Mapt, Snap25, Snca and protection against Aβ oligomers in males, downregulation of DNM1, Mapt and Snca in females	
	miR-4723		Snap25			Downregulation of Snap25 in males	
	miR-149		Creb1			Increase of Creb1 in males	
	miR-200b,miR-200c	Mimics	S6K1	Liposomes	Primary neuronal cells and SH-SY5Y cells	Suppression of cytotoxic damage caused by Aβ	[53]
	miR-200b/c			Lipid nanoparticle	C57BL/6J and Tg2576 mice	Defensive effect against Aβ-induced toxicity	
	miR-31	Mimics	BACE1	Cell transfection	HT-22 cells	Decrease in BACE1 mRNA levels	[54]
			APP, BACE1	Lentivirus	3xTg-AD mice	Amelioration of deficits in memory, reduced anxiety and could prevent the progression of cognitive decline	
	miR-16	Mimics	APP	Liposomes	Neuroblastoma2a, NIH3T3 murine cells	Reduction of APP protein level	[55]
				Infusion	SAMP and SAMR1 mice		
	miR-592	Inhibitor	KIAA0319	Liposomes	Astrocytes	Promotion of astrocytes viability, decrease oxidative stress injury in ASTs, reduction in C-keap1 expression, activation of Keap1/Nrf2/ARE signaling pathway	[57]
	miR-101	Mimics	APP	Liposomes	HeLa cells	Reduction of APP levels	[58]
				Liposomes	Hela, U373, PC12 cells		
	miR-206	Inhibitor	BDNF	Cell transfection	Neuro2a cells	Increase of BDNF protein levels	[56]
					Primary hippocampal neurons	Increase of dendritic spine density, enhanced hippocampal neurogenesis	
				Intranasal administration	Mice	Increase DFNF protein levels, enhanced synaptogenesis, and neurogenesis, improvement of memory function	
Parkinson’s Disease (PD)	miR-205	Mimics	LRRK2	Liposomes	Cell lines, primary neuron cultures, and mouse midbrain dopaminergic neurons	Reducted expression of LRRK2 and prevention of neurite outgrowth defects	[62]
	mir30-hSNCA	Mimics	SNCA	AAV2/8 (unilateral SN injection)	Rats	Protection against the deficit in the forelimb and partial protection against TH-IR cell loss in the SN	[63]
	miR-30e	Mimics	NLRP3	miR-30e agomir injected using a catheter	MPTP-treated PD mice	Reduction of neuroinflammation and lowered expression of α-synuclein	[64]
	miR-124	Mimics	BIM	Exogenous delivery	MPTP-treated PD mice	Reduction of autophagy, apoptosis and lysosomal exhausting	[65]
	miR-124	Mimics	BIM	Cell transfection	MPP+-intoxicated SH-SY5Y cells	Reduction of autophagy, apoptosis and lysosomal exhausting	[65]
	miR-124	Mimics	Sox9 Jagged1	Cell transfection with NPs	Neural stem cells and neuroblasts	Neuronal maturation and proliferation	[66,67]
	miR-124	Mimics	Sox9 Jagged1	Injection of NPs in the lateral ventricle	(6-OHDA) lesioned mice	Neuronal migration to the olfactory bulb and damaged striatum	[66,67]
	miR-181a	Mimics	Regulation of the p38 MAPK and JNK signaling pathways	Liposomes	Human MPP+-induced SK-N-SH neuroblastoma cells	Diminution of the autophagy process and apoptosis	[68]
	miR-185	Mimics	Regulation of the AMPK/mTOR signaling pathway	Liposomes	MPTP-treated SH-SY5Y cells	Diminution of the autophagy process and apoptosis	[69]
	miR-183	Mimics	OSMR	Liposomes	SN neurons from MPTP-treated PD mice	Apoptosis of SN neurons	[70]
	miR-155	-	MHCII	-	Mice with an entire deletion of the miR-155	Regulation of the inflammation and neurodegeneration	[71]
	miR-155	Mimics	MHCII	Oligonucleotide treatment	Microglial cells	Inflammatory response associated with α-synuclein	[71]
	miR-7	Mimics	NLPR3	Stereotactical injection into the striatum	MPTP-treated PD mice	Regulation of the NLPR3 inflammasome activation and reduction in the degeneration of dopaminergic neurons	[72]
	miR-494-3p	Inhibitor	SIRT3	Liposomes	MPP+-induced SH-SY5Y cells	Decrease of neurotoxicity and motor deterioration	[73]
	miR-9-5p	Inhibitor	SIRT1	Liposomes	MPP+-induced SH-SY5Y cells	Decrease of neurotoxicity, apoptosis, oxidative stress and inflammation	[75]
	miR-96	Inhibitor	CACNG5	Injection of inhibitor	MPTP-treated PD mice	Inhibition of both apoptosis and iNOS by blocking the MAPK signaling pathway	[76]
	miR-96	Inhibitor	CACNG5	Liposomes	SH-SY5Y cells	Increment of nigral cells	[76]
	miR-216a	Mimics	Bax	Liposomes	MPP+-treated SK-SY5Y neuroblastoma cells	Decrease of neurotoxicity and apoptosis	[77]
Huntington’s Disease (HD)	mi306.12v16G	Artificial miRNA	HTT	AAV2/1 (bilateral injection in the striata)	Transgenic HD mice	Absence of preferential silencing	[84]
	mi268.5	Artificial miRNA	HTT	AAV2/1 (bilateral injection in the striata)	Transgenic HD mice	Targeting of the mutant allele	[84]
	miR-27a	Mimics	MDR-1	Cell transfection	Derived neuronal stem cells	Reduction in the accumulation of mHTT	[85]
	miR-196a	Mimics	HTT	Cell transfection	Human embryonic renal cells and neuroblastoma cells of mice	Decrease in the expression of mHTT	[86]
	miR-196a	Mimics	HTT	Lentiviral transgenesis	Transgenic HD mice	Decrease in the expression of mHTT	[86]
	miR-196a	Mimics	HTT	Lentiviral infection	HD-iPSCs	Decrease in the expression of mHTT	[86]
	miR-196a	Mimics	RANBP10	Liposomes	Primary cortical neurons	Differentiation and development of neuronal cells	[87]
	miR-196a	Mimics	RANBP10	The hsa-miR-196a-2 precursor was in the transgenic mice	Transgenic HD mice	Differentiation and development of neuronal cells	[87]
	miR-132	Mimics	MeCP2	AAV9 (intracranial injection)	Transgenic HD mice	Symptom improvement and life extension	[88]
	miR-124	Mimics	RE1 silencing transcription factor	Exosomal delivery	Transgenic HD mice	Reduced expression of the RE1 silencing transcription factor	[89]
	miR-124	Mimics	SOX9	Striatal injection	Transgenic HD mice	Increase of neurogenesis in the striatum and cortex and neuronal survival	[90]
	miR-22	Mimics	MAPK14 Trp53inp1 Rcor1 HDAC4 Rgs2	Lentiviral infection	Cultures of primary cortical and striatal neurons	Apoptosis inhibition, decrease in the cumulation of HTT and targeting of different genes	[91]
	miHTT	Artificial miRNA	HTT	AAV9 (striatal and cortical injection)	Transgenic HD sheep	Decrease in the cumulation of mHTT in the neostriatum	[92]
	miHTT	Artificial miRNA	HTT	AAV5 (intracerebral administration)	Lentiviral HD rats	Reduction in the accumulation of mHTT	[93]
	miHTT	Artificial miRNA	HTT	AAV5 (intracranial administration)	Transgenic HD minipig	Decrease in the cumulation of mHTT	[94]
	miHTT	Artificial miRNA	HTT	AAV5 (intracranial injection)	Transgenic HD mice	Decrease in the cumulation of HTT, weight gain, longer life expectancy, and functional improvement	[95]
	miHTT	Artificial miRNA	HTT	AAV5 (intrastriatal administration)	Hu128/21 mouse model of HD	Suppression of HTT	[96]
Amyotrophic Lateral Sclerosis (ALS)	miR-155	Inhibitor (antagomir)	Src homology-2 domain-containing SHIP1 and suppressor of cytokine signaling-1	ICV	SOD1^G93A^ mice	Prolonged survival rate, slower disease progress	[105]
	miR-29a	Inhibitor (antagomir)	NAV3, BACE1	ICV	SOD1^G93A^ mice	Increased lifespan	[106]
	amiRSOD1	Artificial miRNA	SOD1	AAV9	SOD1^G93A^ mice	Extended survival, delayed hindlimb paralysis, reduction of mutant human SOD1	[108]
	miC	Artificial miRNA	C9orf72	AAV5	HEK293T cells and iPSC-derived neurons	Silencing of C9orf72	[104]
	miR-SOD1	Artificial miRNA	SOD1	rAAVrh10	Marmoset	Silencing of SOD1	[109]
	miR-SOD1	Artificial miRNA	SOD1	AAVrh10 (Intralingual and Intrapleural injection)	SOD1^G93A^ mice	Delayed disease onset, prolonged survival, improved breathing	[110]
	miR-SOD1	Artificial miRNA	SOD1	rAAVrh10	SOD1^G93A^ mice	Delayed disease onset, extended survival	[111]
Friedreich’s Ataxia (FRDA)	miR-886-3p	Inhibitor	FXN	Cell transfection	Fibroblast	Increase frataxin levels	[117]
	miR-124	Mimics	FXN	Cell transfection	HEK-293	Down-regulation of FRDA-3-UTR haplotype	[4]
Spinal Muscular Atrophy (SMA)	miR-146a	Inhibitor	Chodl	Endo-Porter peptide	iPSC-derived motor neurons	Restoration of neurite length	[125]
	miR-431	Inhibitor	Chodl	Cell transfection	Motor neurons	Restoration of neurite length	[126]
	miR-206	Mimics	HDAC4	Liposomes	Myoblast cell line C2C12	FGFBP1 transcriptional up-regulation	[127]
	miR-206	Mimics	HDAC4	ICV	Pups and mice	Down-regulation of NCX2, reduction in the severity and progress of SMA pathology	[128]
Frontotemporal Dementia (FTD)	miR-29b	Mimics	GRN	Liposomes	NIH3T3 and Hek293 cells	Regulation of the expression of hPGRN	[138]
	miR-124	Mimics	Gria2 and Gria4	AAV-GFP injection	tTA: CHMP2B^Intron5^ mice	Reduction of the expression of Gria2, Gria4 and improvement of social behavior	[139]
	miR-132/212	Mimics	TMEM106B	Liposomes	HEK293 and SHSY5Y cells	Reduction of TMEM106B mRNA levels	[140]
	miR-107	Mimics	GRN	Liposomes	H4 and HeLa cells	Reduction of GRN expression	[141]
	miR-659	Mimics	GRN	Liposomes	M17 and N2A neuroblastoma cells	Reduction of GRN expression	[142]

## References

[1] Michlewski G., Cáceres J.F. (2019). Post-transcriptional control of miRNA biogenesis. RNA.

[2] Sun P., Liu D.Z., Jickling G.C., Sharp F.R., Yin K.J. (2018). MicroRNA-based therapeutics in central nervous system injuries. J. Cereb. Blood Flow Metab..

[3] Paul S., Reyes P.R., Garza B.S., Sharma A. (2020). MicroRNAs and Child Neuropsychiatric Disorders: A Brief Review. Neurochem. Res..

[4] Bandiera S., Cartault F., Jannot A.S., Hatem E., Girard M., Rifai L., Loiseau C., Munnich A., Lyonnet S., Henrion-Caude A. (2013). Genetic Variations Creating MicroRNA Target Sites in the FXN 3′-UTR Affect Frataxin Expression in Friedreich Ataxia. PLoS ONE.

[5] Sharma S., Lu H.C. (2018). microRNAs in Neurodegeneration: Current Findings and Potential Impacts. J. Alzheimer’s Dis. Park..

[6] Santhekadur P.K., Kumar D.P. (2020). RISC assembly and post-transcriptional gene regulation in Hepatocellular Carcinoma. Genes Dis..

[7] Gupta S., Kulhara P. (2010). What is schizophrenia: A neurodevelopmental or neurodegenerative disorder or a combination of both A critical analysis. Indian J. Psychiatry.

[8] Kim J., Mook-Jung I. (2015). Special issue on neurodegenerative diseases and their therapeutic approaches. Exp. Mol. Med..

[9] Kovacs G.G. (2019). Molecular pathology of neurodegenerative diseases: Principles and practice. J. Clin. Pathol..

[10] Qiu L., Tan E.K., Zeng L. (2015). microRNAs and Neurodegenerative Diseases. Adv. Exp. Med. Biol..

[11] Walayat A., Yang M., Xiao D., Sharad S., Kapur S. (2019). Therapeutic Implication of miRNA in Human Disease. Antisense Therapy.

[12] Christopher A.F., Kaur R.P., Kaur G., Kaur A., Gupta V., Bansal P. (2016). MicroRNA therapeutics: Discovering novel targets and developing specific therapy. Perspect. Clin. Res..

[13] Liu E.Y., Cali C.P., Lee E.B. (2017). RNA metabolism in neurodegenerative disease. DMM Dis. Model. Mech..

[14] Bernardo B.C., Ooi J.Y.Y., Lin R.C.Y., McMullen J.R. (2015). miRNA therapeutics: A new class of drugs with potential therapeutic applications in the heart. Future Med. Chem..

[15] Leclercq M., Diallo A.B., Blanchette M. (2017). Prediction of human miRNA target genes using computationally reconstructed ancestral mammalian sequences. Nucleic Acids Res..

[16] Bajan S., Hutvagner G. (2020). RNA-Based Therapeutics: From Antisense Oligonucleotides to miRNAs. Cells.

[17] Campbell K., Booth S.A. (2015). MicroRNA in neurodegenerative drug discovery: The way forward?. Expert Opin. Drug Discov..

[18] Goldgraben M.A., Russell R., Rueda O.M., Caldas C., Git A. (2016). Double-stranded microRNA mimics can induce length-and passenger strand-dependent effects in a cell type-specific manner. RNA.

[19] Lin H.M., Nikolic I., Yang J., Castillo L., Deng N., Chan C.L., Yeung N.K., Dodson E., Elsworth B., Spielman C. (2018). MicroRNAs as potential therapeutics to enhance chemosensitivity in advanced prostate cancer. Sci. Rep..

[20] Tang L., Chen H.Y., Hao N.B., Tang B., Guo H., Yong X., Dong H., Yang S.M. (2017). microRNA inhibitors: Natural and artificial sequestration of microRNA. Cancer Lett..

[21] Lima J.F., Cerqueira L., Figueiredo C., Oliveira C., Azevedo N.F. (2018). Anti-miRNA oligonucleotides: A comprehensive guide for design. RNA Biol..

[22] Ebert M.S., Sharp P.A. (2010). MicroRNA sponges: Progress and possibilities. RNA.

[23] Simonson B., Das S. (2015). MicroRNA Therapeutics: The Next Magic Bullet?. Mini Rev. Med. Chem..

[24] Pereira D.M., Rodrigues P.M., Borralho P.M., Rodrigues C.M.P. (2013). Delivering the promise of miRNA cancer therapeutics. Drug Discov. Today.

[25] Sun X., Guo Q., Wei W., Robertson S., Yuan Y., Luo X. (2019). Current progress on microRNA-based gene delivery in the treatment of osteoporosis and osteoporotic fracture. Int. J. Endocrinol..

[26] Yang N. (2015). An overview of viral and nonviral delivery systems for microRNA. Int. J. Pharm. Investig..

[27] Myoung S., Kasinski A.L. (2019). Strategies for Safe and Targeted Delivery of MicroRNA Therapeutics. MicroRNAs in Diseases and Disorders: Emerging Therapeutic Targets.

[28] Ojala D.S., Amara D.P., Schaffer D.V. (2015). Adeno-associated virus vectors and neurological gene therapy. Neuroscientist.

[29] Lee S.W.L., Paoletti C., Campisi M., Osaki T., Adriani G., Kamm R.D., Mattu C., Chiono V. (2019). MicroRNA delivery through nanoparticles. J. Control. Release.

[30] Zhang Y., Wang Z., Gemeinhart R.A. (2013). Progress in microRNA delivery. J. Control. Release.

[31] Carter M., Shieh J. (2015). Gene Delivery Strategies. Guide to Research Techniques in Neuroscience.

[32] Wen M.M. (2016). Getting miRNA therapeutics into the target cells for neurodegenerative diseases: A mini-review. Front. Mol. Neurosci..

[33] Wang H., Jiang Y., Peng H., Chen Y., Zhu P., Huang Y. (2015). Recent progress in microRNA delivery for cancer therapy by non-viral synthetic vectors. Adv. Drug Deliv. Rev..

[34] Magen I., Hornstein E. (2014). Oligonucleotide-based therapy for neurodegenerative diseases. Brain Res..

[35] Campani V., De Rosa G., Misso G., RZarone M., Grimaldi A. (2016). Lipid Nanoparticles to Deliver miRNA in Cancer. Curr. Pharm. Biotechnol..

[36] Estanqueiro M., Vasconcelos H., Lobo J.M.S., Amaral H., Grumezescu A.M. (2018). Delivering miRNA modulators for cancer treatment. Drug Targeting and Stimuli Sensitive Drug Delivery Systems.

[37] Huynh N., VonMoss L., Smith D., Rahman I., Felemban M.F., Zuo J., Rody W.J., McHugh K.P., Holliday L.S. (2016). Characterization of regulatory extracellular vesicles from osteoclasts. J. Dent. Res..

[38] Khanahmadi M., Farhud D.D., Malmir M. (2015). Genetic of Alzheimer’s disease: A narrative review article. Iran. J. Public Health.

[39] Chen J.J., Zhao B., Zhao J., Li S. (2017). Potential Roles of Exosomal MicroRNAs as Diagnostic Biomarkers and Therapeutic Application in Alzheimer’s Disease. Neural Plast..

[40] Bekris L.M., Leverenz J.B. (2015). The biomarker and therapeutic potential of miRNA in Alzheimer’s disease. Neurodegener. Dis. Manag..

[41] Angelucci F., Cechova K., Valis M., Kuca K., Zhang B., Hort J. (2019). MicroRNAs in Alzheimer’s disease: Diagnostic markers or therapeutic agents?. Front. Pharmacol..

[42] Parsi S., Smith P.Y., Goupil C., Dorval V., Hébert S.S. (2015). Preclinical evaluation of miR-15/107 family members as multifactorial drug targets for Alzheimer’s disease. Mol. Ther. Nucleic Acids.

[43] Smith P.Y., Hernandez-Rapp J., Jolivette F., Lecours C., Bisht K., Goupil C., Dorval V., Parsi S., Morin F., Planel E. (2015). MiR-132/212 deficiency impairs tau metabolism and promotes pathological aggregation in vivo. Hum. Mol. Genet..

[44] Salta E., Sierksma A., Vanden Eynden E., De Strooper B. (2016). miR-132 loss de-represses ITPKB and aggravates amyloid and TAU pathology in Alzheimer’s brain. EMBO Mol. Med..

[45] Fang M., Wang J., Zhang X., Geng Y., Hu Z., Rudd J.A., Ling S., Chen W., Han S. (2012). The miR-124 regulates the expression of BACE1/β-secretase correlated with cell death in Alzheimer’s disease. Toxicol. Lett..

[46] Cao F., Liu Z., Sun G. (2020). Diagnostic value of miR-193a-3p in Alzheimer’s disease and miR-193a-3p attenuates amyloid-β induced neurotoxicity by targeting PTEN. Exp. Gerontol..

[47] Sun L., Zhang T., Xiu W., Cao W., He M., Sun W., Zhao W. (2020). MiR-107 overexpression attenuates neurotoxicity induced by 6-hydroxydopamine both in vitro and in vivo. Chem. Biol. Interact..

[48] He B., Chen W., Zeng J., Tong W., Zheng P. (2020). MicroRNA-326 decreases tau phosphorylation and neuron apoptosis through inhibition of the JNK signaling pathway by targeting VAV1 in Alzheimer’s disease. J. Cell. Physiol..

[49] Zovoilis A., Agbemenyah H.Y., Agis-Balboa R.C., Stilling R.M., Edbauer D., Rao P., Farinelli L., Delalle I., Schmitt A., Falkai P. (2011). MicroRNA-34c is a novel target to treat dementias. EMBO J..

[50] Shi Z., Zhang K., Zhou H., Jiang L., Xie B., Wang R., Xia W., Yin Y., Gao Z., Cui D. (2020). Increased miR-34c mediates synaptic deficits by targeting synaptotagmin 1 through ROS-JNK-p53 pathway in Alzheimer’s Disease. Aging Cell.

[51] Lee K., Kim H., An K., Kwon O.B., Park S., Cha J.H., Kim M.H., Lee Y., Kim J.H., Cho K. (2016). Replenishment of microRNA-188-5p restores the synaptic and cognitive deficits in 5XFAD Mouse Model of Alzheimer’s Disease. Sci. Rep..

[52] Zolochevska O., Taglialatela G. (2020). Selected microRNAs Increase Synaptic Resilience to the Damaging Binding of the Alzheimer’s Disease Amyloid Beta Oligomers. Mol. Neurobiol..

[53] Higaki S., Muramatsu M., Matsuda A., Matsumoto K., Satoh J.I., Michikawa M., Niida S. (2018). Defensive effect of microRNA-200b/c against amyloid-beta peptide-induced toxicity in Alzheimer’s disease models. PLoS ONE.

[54] Barros-Viegas A.T., Carmona V., Ferreiro E., Guedes J., Cardoso A.M., Cunha P., Pereira de Almeida L., Resende de Oliveira C., Pedro de Magalhães J., Peça J. (2020). miRNA-31 Improves Cognition and Abolishes Amyloid-β Pathology by Targeting APP and BACE1 in an Animal Model of Alzheimer’s Disease. Mol. Ther. Nucleic Acids.

[55] Liu W., Liu C., Zhu J., Shu P., Yin B., Gong Y., Qiang B., Yuan J., Peng X. (2012). MicroRNA-16 targets amyloid precursor protein to potentially modulate Alzheimer’s-associated pathogenesis in SAMP8 mice. Neurobiol. Aging.

[56] Lee S.T., Chu K., Jung K.H., Kim J.H., Huh J.Y., Yoon H., Park D.K., Lim J.Y., Kim J.M., Jeon D. (2012). MiR-206 regulates brain-derived neurotrophic factor in Alzheimer disease model. Ann. Neurol..

[57] Wu G.D., Li Z.H., Li X., Zheng T., Zhang D.K. (2020). microRNA-592 blockade inhibits oxidative stress injury in Alzheimer’s disease astrocytes via the KIAA0319-mediated Keap1/Nrf2/ARE signaling pathway. Exp. Neurol..

[58] Long J.M., Lahiri D.K. (2011). MicroRNA-101 downregulates Alzheimer’s amyloid-β precursor protein levels in human cell cultures and is differentially expressed. Biochem. Biophys. Res. Commun..

[59] Aarsland D., Creese B., Politis M., Chaudhuri K.R., Ffytche D.H., Weintraub D., Ballard C. (2017). Cognitive decline in Parkinson disease. Nat. Rev. Neurol..

[60] Blauwendraat C., Reed X., Krohn L., Heilbron K., Bandres-Ciga S., Tan M., Gibbs J.R., Hernandez D.G., Kumaran R., Langston R. (2020). Genetic modifiers of risk and age at onset in GBA associated Parkinson’s disease and Lewy body dementia. Brain.

[61] Blauwendraat C., Reed X., Kia D.A., Gan-Or Z., Lesage S., Pihlstrøm L., Guerreiro R., Gibbs J.R., Sabir M., Ahmed S. (2018). Frequency of loss of function variants in LRRK2 in Parkinson disease. JAMA Neurol..

[62] Cho H.J., Liu G., Jin S.M., Parisiadou L., Xie C., Yu J., Sun L., Ma B., Ding J., Vancraenenbroeck R. (2013). Microrna-205 regulates the expression of parkinson’s disease-related leucine-rich repeat kinase 2 protein. Hum. Mol. Genet..

[63] Khodr C.E., Becerra A., Han Y., Bohn M.C. (2014). Targeting alpha-synuclein with a microRNA-embedded silencing vector in the rat substantia nigra: Positive and negative effects. Brain Res..

[64] Li D., Yang H., Ma J., Luo S., Chen S., Gu Q. (2018). MicroRNA-30e regulates neuroinflammation in MPTP model of Parkinson’s disease by targeting Nlrp3. Hum. Cell.

[65] Wang H., Ye Y., Zhu Z., Mo L., Lin C., Wang Q., Wang H., Gong X., He X., Lu G. (2016). MiR-124 regulates apoptosis and autophagy process in MPTP model of Parkinson’s disease by targeting to bim. Brain Pathol..

[66] Saraiva C., Ferreira L., Bernardino L. (2016). Traceable microRNA-124 loaded nanoparticles as a new promising therapeutic tool for Parkinson’s disease. Neurogenesis.

[67] Saraiva C., Paiva J., Santos T., Ferreira L., Bernardino L. (2016). MicroRNA-124 loaded nanoparticles enhance brain repair in Parkinson’s disease. J. Control. Release.

[68] Liu Y., Song Y., Zhu X. (2017). MicroRNA-181a regulates apoptosis and autophagy process in Parkinson’s disease by inhibiting p38 mitogen-activated protein kinase (MAPK)/c-Jun N-terminal kinases (JNK) signaling pathways. Med. Sci. Monit..

[69] Wen Z., Zhang J., Tang P., Tu N., Wang K., Wu G. (2018). Overexpression of miR-185 inhibits autophagy and apoptosis of dopaminergic neurons by regulating the AMPK/mTOR signaling pathway in Parkinson’s disease. Mol. Med. Rep..

[70] Gao J.X., Li Y., Wang S.N., Chen X.C., Lin L.L., Zhang H. (2019). Overexpression of microRNA-183 promotes apoptosis of substantia nigra neurons via the inhibition of OSMR in a mouse model of Parkinson’s disease. Int. J. Mol. Med..

[71] Thome A.D., Harms A.S., Volpicelli-Daley L.A., Standaert D.G. (2016). MicroRNA-155 regulates alpha-synuclein-induced inflammatory responses in models of Parkinson disease. J. Neurosci..

[72] Zhou Y., Lu M., Du R.-H., Qiao C., Jiang C.-Y., Zhang K.-Z., Ding J.-H., Hu G. (2016). MicroRNA-7 targets Nod-like receptor protein 3 inflammasome to modulate neuroinflammation in the pathogenesis of Parkinson’s disease. Mol. Neurodegener..

[73] Geng L., Zhang T., Liu W., Chen Y. (2018). miR-494-3p modulates the progression of in vitro and in vivo Parkinson’s disease models by targeting SIRT3. Neurosci. Lett..

[74] Ansari A., Rahman M.S., Saha S.K., Saikot F.K., Deep A., Kim K.H. (2017). Function of the SIRT3 mitochondrial deacetylase in cellular physiology, cancer, and neurodegenerative disease. Aging Cell.

[75] Wang Z., Sun L., Jia K., Wang H., Wang X. (2019). miR-9-5p modulates the progression of Parkinson’s disease by targeting SIRT1. Neurosci. Lett..

[76] Dong Y., Han L.L., Xu Z.X. (2018). Suppressed microRNA-96 inhibits iNOS expression and dopaminergic neuron apoptosis through inactivating the MAPK signaling pathway by targeting CACNG5 in mice with Parkinson’s disease. Mol. Med..

[77] Yang X., Zhang M., Wei M., Wang A., Deng Y., Cao H. (2020). MicroRNA-216a inhibits neuronal apoptosis in a cellular Parkinson’s disease model by targeting Bax. Metab. Brain Dis..

[78] Roos R.A.C. (2010). Huntington’s disease: A clinical review. Orphanet J. Rare Dis..

[79] Nopoulos P.C. (2016). Huntington disease: A single-gene degenerative disorder of the striatum. Dialogues Clin. Neurosci..

[80] McColgan P., Tabrizi S.J. (2018). Huntington’s disease: A clinical review. Eur. J. Neurol..

[81] Zhang Y., Friedlander R.M. (2011). Using non-coding small RNAs to develop therapies for Huntington’s disease. Gene Ther..

[82] McBride J.L., Pitzer M.R., Boudreau R.L., Dufour B., Hobbs T., Ojeda S.R., Davidson B.L. (2011). Preclinical safety of RNAi-mediated HTT suppression in the rhesus macaque as a potential therapy for Huntington’s disease. Mol. Ther..

[83] Boudreau R.L., McBride J.L., Martins I., Shen S., Xing Y., Carter B.J., Davidson B.L. (2009). Nonallele-specific silencing of mutant and wild-type huntingtin demonstrates therapeutic efficacy in Huntington’s disease mice. Mol. Ther..

[84] Monteys A.M., Wilson M.J., Boudreau R.L., Spengler R.M., Davidson B.L. (2015). Artificial miRNAs targeting mutant huntingtin show preferential silencing in vitro and in vivo. Mol. Ther. Nucleic Acids.

[85] Ban J.J., Chung J.Y., Lee M., Im W., Kim M. (2017). MicroRNA-27a reduces mutant hutingtin aggregation in an in vitro model of Huntington’s disease. Biochem. Biophys. Res. Commun..

[86] Cheng P.H., Li C.L., Chang Y.F., Tsai S.J., Lai Y.Y., Chan A.W.S., Chen C.M., Yang S.H. (2013). MiR-196a ameliorates phenotypes of huntington disease in cell, transgenic mouse, and induced pluripotent stem cell models. Am. J. Hum. Genet..

[87] Her L.S., Mao S.H., Chang C.Y., Cheng P.H., Chang Y.F., Yang H.I., Chen C.M., Yang S.H. (2017). miR-196a enhances neuronal morphology through suppressing RANBP10 to provide neuroprotection in Huntington’s disease. Theranostics.

[88] Fukuoka M., Takahashi M., Fujita H., Chiyo T., Popiel H.A., Watanabe S., Furuya H., Murata M., Wada K., Okada T. (2018). Supplemental Treatment for Huntington’s Disease with miR-132 that Is Deficient in Huntington’s Disease Brain. Mol. Ther. Nucleic Acids.

[89] Lee S.-T., Im W., Ban J.-J., Lee M., Jung K.-H., Lee S.K., Chu K., Kim M. (2017). Exosome-Based Delivery of miR-124 in a Huntington’s Disease Model. J. Mov. Disord..

[90] Liu T., Im W., Mook-Jung I., Kim M. (2015). MicroRNA-124 slows down the progression of huntington’s disease by promoting neurogenesis in the striatum. Neural Regen. Res..

[91] Jovicic A., Zaldivar Jolissaint J.F., Moser R., Silva Santos M.D., Luthi-Carter R. (2013). MicroRNA-22 (miR-22) Overexpression Is Neuroprotective via General Anti-Apoptotic Effects and May also Target Specific Huntington’s Disease-Related Mechanisms. PLoS ONE.

[92] Pfister E.L., Dinardo N., Mondo E., Borel F., Conroy F., Fraser C., Gernoux G., Han X., Hu D., Johnson E. (2018). Artificial miRNAs Reduce Human Mutant Huntingtin Throughout the Striatum in a Transgenic Sheep Model of Huntington’s Disease. Hum. Gene Ther..

[93] Miniarikova J., Zimmer V., Martier R., Brouwers C.C., Pythoud C., Richetin K., Rey M., Lubelski J., Evers M.M., Van Deventer S.J. (2017). AAV5-miHTT gene therapy demonstrates suppression of mutant huntingtin aggregation and neuronal dysfunction in a rat model of Huntington’s disease. Gene Ther..

[94] Evers M.M., Miniarikova J., Juhas S., Vallès A., Bohuslavova B., Juhasova J., Skalnikova H.K., Vodicka P., Valekova I., Brouwers C. (2018). AAV5-miHTT Gene Therapy Demonstrates Broad Distribution and Strong Human Mutant Huntingtin Lowering in a Huntington’s Disease Minipig Model. Mol. Ther..

[95] Spronck E.A., Brouwers C.C., Vallès A., de Haan M., Petry H., van Deventer S.J., Konstantinova P., Evers M.M. (2019). AAV5-miHTT Gene Therapy Demonstrates Sustained Huntingtin Lowering and Functional Improvement in Huntington Disease Mouse Models. Mol. Ther. Methods Clin. Dev..

[96] Caron N.S., Southwell A.L., Brouwers C.C., Cengio L.D., Xie Y., Black H.F., Anderson L.M., Ko S., Zhu X., Van Deventer S.J. (2020). Potent and sustained huntingtin lowering via AAV5 encoding miRNA preserves striatal volume and cognitive function in a humanized mouse model of Huntington disease. Nucleic Acids Res..

[97] Brown R.H., Al-Chalabi A. (2017). Amyotrophic Lateral Sclerosis. N. Engl. J. Med..

[98] Van Es M.A., Hardiman O., Chio A., Al-Chalabi A., Pasterkamp R.J., Veldink J.H., van den Berg L.H. (2017). Amyotrophic lateral sclerosis. Lancet.

[99] Martin S., Al Khleifat A., Al-Chalabi A. (2017). What causes amyotrophic lateral sclerosis?. F1000Research.

[100] De Felice B., Guida M., Guida M., Coppola C., De Mieri G., Cotrufo R. (2012). A miRNA signature in leukocytes from sporadic amyotrophic lateral sclerosis. Gene.

[101] Ricci C., Marzocchi C., Battistini S. (2018). MicroRNAs as Biomarkers in Amyotrophic Lateral Sclerosis. Cells.

[102] Kovanda A., Leonardis L., Zidar J., Koritnik B., Dolenc-Groselj L., Ristic Kovacic S., Curk T., Rogelj B. (2018). Differential expression of microRNAs and other small RNAs in muscle tissue of patients with ALS and healthy age-matched controls. Sci. Rep..

[103] Philips T., Rothstein J.D. (2015). Rodent Models of Amyotrophic Lateral Sclerosis. Curr. Protoc. Pharmacol..

[104] Martier R., Liefhebber J.M., Miniarikova J., van der Zon T., Snapper J., Kolder I., Petry H., van Deventer S.J., Evers M.M., Konstantinova P. (2019). Artificial MicroRNAs Targeting C9orf72 Can Reduce Accumulation of Intra-nuclear Transcripts in ALS and FTD Patients. Mol. Ther. Nucleic Acids.

[105] Koval E.D., Shaner C., Zhang P., du Maine X., Fischer K., Tay J., Chau B.N., Wu G.F., Miller T.M. (2013). Method for widespread microRNA-155 inhibition prolongs survival in ALS-model mice. Hum. Mol. Genet..

[106] Nolan K., Mitchem M.R., Jimenez-Mateos E.M., Henshall D.C., Concannon C.G., Prehn J.H.M. (2014). Increased Expression of MicroRNA-29a in ALS Mice: Functional Analysis of Its Inhibition. J. Mol. Neurosci..

[107] Shioya M., Obayashi S., Tabunoki H., Arima K., Saito Y., Ishida T., Satoh J. (2010). Aberrant microRNA expression in the brains of neurodegenerative diseases: miR-29a decreased in Alzheimer disease brains targets neurone navigator 3. Neuropathol. Appl. Neurobiol..

[108] Stoica L., Todeasa S.H., Cabrera G.T., Salameh J.S., ElMallah M.K., Mueller C., Brown Jr R.H., Sena-Esteves M. (2016). Adeno-associated virus–delivered artificial microRNA extends survival and delays paralysis in an amyotrophic lateral sclerosis mouse model. Ann. Neurol..

[109] Borel F., Gernoux G., Cardozo B., Metterville J.P., Cabrera G.T., Song L., Su Q., Gao G.P., Elmallah M.K., Brown R.H. (2016). Therapeutic rAAVrh10 Mediated SOD1 Silencing in Adult SOD1(G93A) Mice and Nonhuman Primates. Hum. Gene Ther..

[110] Keeler A.M., Zieger M., Semple C., Pucci L., Veinbachs A., Brown R.H., Mueller C., ElMallah M.K. (2020). Intralingual and Intrapleural AAV Gene Therapy Prolongs Survival in a SOD1 ALS Mouse Model. Mol. Ther. Methods Clin. Dev..

[111] Li D., Liu C., Yang C., Wang D., Wu D., Qi Y., Su Q., Gao G., Xu Z., Guo Y. (2017). Slow Intrathecal Injection of rAAVrh10 Enhances its Transduction of Spinal Cord and Therapeutic Efficacy in a Mutant SOD1 Model of ALS. Neuroscience.

[112] La Rosa P., Bertini E.S., Piemonte F. (2020). The NRF2 signaling network defines clinical biomarkers and therapeutic opportunity in Friedreich’s Ataxia. Int. J. Mol. Sci..

[113] Dantham S., Srivastava A.K., Gulati S., Rajeswari M.R. (2018). Differentially Regulated Cell-Free MicroRNAs in the Plasma of Friedreich’s Ataxia Patients and Their Association with Disease Pathology. Neuropediatrics.

[114] Pandolfo M. (2009). Friedreich ataxia: The clinical picture. J. Neurol..

[115] Koeppen A.H. (2011). Friedreich’s ataxia: Pathology, pathogenesis, and molecular genetics. J. Neurol. Sci..

[116] Harding A.E. (1981). Friedreich’s ataxia: A clinical and genetic study of 90 families with an analysis of early diagnostic criteria and intrafamilial clustering of clinical features. Brain.

[117] Mahishi L.H., Hart R.P., Lynch D.R., Ratan R.R. (2012). miR-886-3p levels are elevated in Friedreich ataxia. J. Neurosci..

[118] Shen J., Zhou W., Bi N., Song Y.-M., Zhang F.-Q., Zhan Q.-M., Wang L.-H. (2018). MicroRNA-886-3P functions as a tumor suppressor in small cell lung cancer. Cancer Biol. Ther..

[119] Groen E.J.N., Talbot K., Gillingwater T.H. (2018). Advances in therapy for spinal muscular atrophy: Promises and challenges. Nat. Rev. Neurol..

[120] Schellino R., Boido M., Vercelli A. (2019). JNK Signaling Pathway Involvement in Spinal Cord Neuron Development and Death. Cells.

[121] Haramati S., Chapnik E., Sztainberg Y., Eilam R., Zwang R., Gershoni N., McGlinn E., Heiser P.W., Wills A.-M., Wirguin I. (2010). miRNA malfunction causes spinal motor neuron disease. Proc. Natl. Acad. Sci. USA.

[122] Magri F., Vanoli F., Corti S. (2018). miRNA in spinal muscular atrophy pathogenesis and therapy. J. Cell. Mol. Med..

[123] Luchetti A., Ciafrè S., Murdocca M., Malgieri A., Masotti A., Sanchez M., Farace M., Novelli G., Sangiuolo F. (2015). A Perturbed MicroRNA Expression Pattern Characterizes Embryonic Neural Stem Cells Derived from a Severe Mouse Model of Spinal Muscular Atrophy (SMA). Int. J. Mol. Sci..

[124] Amin N.D., Bai G., Klug J.R., Bonanomi D., Pankratz M.T., Gifford W.D., Hinckley C.A., Sternfeld M.J., Driscoll S.P., Dominguez B. (2015). Loss of motoneuron-specific microRNA-218 causes systemic neuromuscular failure. Science.

[125] Sison S.L., Patitucci T.N., Seminary E.R., Villalon E., Lorson C.L., Ebert A.D. (2017). Astrocyte-produced miR-146a as a mediator of motor neuron loss in spinal muscular atrophy. Hum. Mol. Genet..

[126] Wertz M.H., Winden K., Neveu P., Ng S.-Y., Ercan E., Sahin M. (2016). Cell-type-specific miR-431 dysregulation in a motor neuron model of spinal muscular atrophy. Hum. Mol. Genet..

[127] Valsecchi V., Boido M., De Amicis E., Piras A., Vercelli A. (2015). Expression of Muscle-Specific MiRNA 206 in the Progression of Disease in a Murine SMA Model. PLoS ONE.

[128] Valsecchi V., Anzilotti S., Serani A., Laudati G., Brancaccio P., Guida N., Cuomo O., Pignataro G., Annunziato L. (2020). miR-206 Reduces the Severity of Motor Neuron Degeneration in the Facial Nuclei of the Brainstem in a Mouse Model of SMA. Mol. Ther..

[129] Greaves C.V., Rohrer J.D. (2019). An update on genetic frontotemporal dementia. J. Neurol..

[130] Olney N.T., Spina S., Miller B.L. (2017). Frontotemporal Dementia. Neurol. Clin..

[131] Goldman J.S., Van Deerlin V.M. (2018). Alzheimer’s Disease and Frontotemporal Dementia: The Current State of Genetics and Genetic Testing Since the Advent of Next-Generation Sequencing. Mol. Diagnosis Ther..

[132] Bennion Callister J., Pickering-Brown S.M. (2014). Pathogenesis/genetics of frontotemporal dementia and how it relates to ALS. Exp. Neurol..

[133] Bott N.T., Radke A., Stephens M.L., Kramer J.H. (2014). Frontotemporal dementia: Diagnosis, deficits and management. Neurodegener. Dis. Manag..

[134] Olszewska D.A., Lonergan R., Fallon E.M., Lynch T. (2016). Genetics of Frontotemporal Dementia. Curr. Neurol. Neurosci. Rep..

[135] Piscopo P., Albani D., Castellano A.E., Forloni G., Confaloni A. (2016). Frontotemporal lobar degeneration and microRNAs. Front. Aging Neurosci..

[136] Rabinovici G.D., Miller B.L. (2010). Frontotemporal lobar degeneration: Epidemiology, pathophysiology, diagnosis and management. CNS Drugs.

[137] Kocerha J., Kouri N., Baker M., Finch N.C., DeJesus-Hernandez M., Gonzalez J., Chidamparam K., Josephs K.A., Boeve B.F., Graff-Radford N.R. (2011). Altered microRNA expression in frontotemporal lobar degeneration with TDP-43 pathology caused by progranulin mutations. BMC Genom..

[138] Jiao J., Herl L.D., Farese R.V., Gao F.-B. (2010). MicroRNA-29b Regulates the Expression Level of Human Progranulin, a Secreted Glycoprotein Implicated in Frontotemporal Dementia. PLoS ONE.

[139] Gascon E., Lynch K., Ruan H., Almeida S., Verheyden J.M., Seeley W.W., Dickson D.W., Petrucelli L., Sun D., Jiao J. (2014). Alterations in microRNA-124 and AMPA receptors contribute to social behavioral deficits in frontotemporal dementia. Nat. Med..

[140] Chen-Plotkin A.S., Unger T.L., Gallagher M.D., Bill E., Kwong L.K., Volpicelli-Daley L., Busch J.I., Akle S., Grossman M., Van Deerlin V. (2012). TMEM106B, the risk gene for frontotemporal dementia, is regulated by the microRNA-132/212 cluster and affects progranulin pathways. J. Neurosci..

[141] Wang W.X., Wilfred B.R., Madathil S.K., Tang G., Hu Y., Dimayuga J., Stromberg A.J., Huang Q., Saatman K.E., Nelson P.T. (2010). miR-107 regulates granulin/progranulin with implications for traumatic brain injury and neurodegenerative disease. Am. J. Pathol..

[142] Rademakers R., Eriksen J.L., Baker M., Robinson T., Ahmed Z., Lincoln S.J., Finch N., Rutherford N.J., Crook R.J., Josephs K.A. (2008). Common variation in the miR-659 binding-site of GRN is a major risk factor for TDP43-positive frontotemporal dementia. Hum. Mol. Genet..

[143] Hashimoto Y., Akiyama Y., Yuasa Y. (2013). Multiple-to-multiple relationships between microRNAs and target genes in gastric cancer. PLoS ONE.

[144] Titze-de-Almeida S.S., Soto-Sánchez C., Fernandez E., Koprich J.B., Brotchie J.M., Titze-de-Almeida R. (2020). The Promise and Challenges of Developing miRNA-Based Therapeutics for Parkinson’s Disease. Cells.

[145] Guo L., Zhang Q., Ma X., Wang J., Liang T. (2017). miRNA and mRNA expression analysis reveals potential sex-biased miRNA expression. Sci. Rep..

[146] Keller A., Rounge T., Backes C., Ludwig N., Gislefoss R., Leidinger P., Langseth H., Meese E. (2017). Sources to variability in circulating human miRNA signatures. RNA Biol..

